# VIPER: a visualisation tool for exploring inheritance inconsistencies in genotyped pedigrees

**DOI:** 10.1186/1471-2105-13-S8-S5

**Published:** 2012-05-18

**Authors:** Trevor Paterson, Martin Graham, Jessie Kennedy, Andy Law

**Affiliations:** 1 The Roslin Institute, Royal (Dick) School of Veterinary Studies, University of Edinburgh, Easter Bush, Midlothian, EH25 9RG, Scotland, UK; 2 Institute for Informatics & Digital Innovation, Edinburgh Napier University, Colinton Road, Edinburgh, EH10 5DT, Scotland, UK

## Abstract

**Background:**

Pedigree genotype datasets are used for analysing genetic inheritance and to map genetic markers and traits. Such datasets consist of hundreds of related animals genotyped for thousands of genetic markers and invariably contain multiple errors in both the pedigree structure and in the associated individual genotype data. These errors manifest as apparent inheritance inconsistencies in the pedigree, and invalidate analyses of marker inheritance patterns across the dataset. Cleaning raw datasets of bad data points (incorrect pedigree relationships, unreliable marker assays, suspect samples, bad genotype results *etc*.) requires expert exploration of the patterns of exposed inconsistencies in the context of the inheritance pedigree. In order to assist this process we are developing VIPER (Visual Pedigree Explorer), a software tool that integrates an inheritance-checking algorithm with a novel space-efficient pedigree visualisation, so that reported inheritance inconsistencies are overlaid on an interactive, navigable representation of the pedigree structure.

**Methods and results:**

This paper describes an evaluation of how VIPER displays the different scales and types of dataset that occur experimentally, with a description of how VIPER's display interface and functionality meet the challenges presented by such data. We examine a range of possible error types found in real and simulated pedigree genotype datasets, demonstrating how these errors are exposed and explored using the VIPER interface and we evaluate the utility and usability of the interface to the domain expert.

Evaluation was performed as a two stage process with the assistance of domain experts (geneticists). The initial evaluation drove the iterative implementation of further features in the software prototype, as required by the users, prior to a final functional evaluation of the pedigree display for exploring the various error types, data scales and structures.

**Conclusions:**

The VIPER display was shown to effectively expose the range of errors found in experimental genotyped pedigrees, allowing users to explore the underlying causes of reported inheritance inconsistencies. This interface will provide the basis for a full data cleaning tool that will allow the user to remove isolated bad data points, and reversibly test the effect of removing suspect genotypes and pedigree relationships.

## Background

Genotyped pedigree data underpins many forms of genetic analyses that are performed by breeders and biologists to identify, map and select economically or biologically important genes or heritable traits. For techniques such as linkage analysis, genotype scores for polymorphic markers distributed across the genome are analysed in the context of the pedigree structure and Mendelian laws of inheritance (i.e. each parent contributing a single allele to the offspring genotype). The statistical algorithms applied in such genetic analyses are critically sensitive to any errors in the data that exhibit as 'inheritance inconsistencies', i.e. patterns of inheritance for alleles that are not consistent with the asserted parent-child relationships recorded in the pedigree. Any such errors must be identified and cleansed from the data before downstream analyses. Error cleansing constitutes a complex, labour-intensive expert task, particularly given the scale of modern genotyping studies, where populations of several thousand animals may be genotyped for tens of thousands of markers.

### Pedigree and genotype data

Roslin Bioinformatics provides the web-based ResSpecies data system for recording and analysing animal pedigree-genotype data [1]. The experimental pedigrees available in ResSpecies, particularly those from the five major farmed species (Chicken, Turkey, Pig, Cow and Sheep) exemplify the variety in structure and scale of study populations with which we deal and these pedigrees have been used (after anonymisation) for the generation of test datasets employed in the evaluation.

ResSpecies pedigrees range in size from 45 to 11000 individuals, but more typical sizes range from 100 to 2500. The structure of each particular pedigree reflects the design of the breeding experiment, e.g. inbreeding versus outbreeding, with the number of generations varying between 2 and 11. Similarly the number of founder animals in each pedigree varies between 2 and 1200; founders may be introduced throughout a breeding program, not only in 'generation 0', and the proportion of founders used in a study varies greatly from 0.3% to 55%. The proportion of males recorded in a pedigree ranges from 0.7% to 94%, whilst the proportion of females varies between 2 and 98%. Some pedigrees, particularly fowl studies, may not record the sex of animals which are not kept for breeding, resulting in up to 98% of individuals unsexed, however, more typically only a few percent of animals are unsexed. Sexing becomes a particular issue when identifying the inheritance pattern of sex-linked markers. The shape of pedigrees (i.e. the number/proportion of individuals per generation) also varies, with some experiments using very few individuals in earlier generations, but generating large numbers in the final generations. The choice of mate selection is also study dependant, with some studies crossing a single individual (often a male) with multiple partners, even across generations. This great variety in pedigree structure differs from typical human pedigrees, and the imbalances, multiple and cross generational pairings, and in some cases the size of families, present additional challenges for a successful pedigree visualisation which would allow the user to trace inheritance patterns from ancestors to descendants and siblings.

Inheritance studies use genotypes scored for any number of detectable genetic markers distributed across the genome of the study organism. A specific marker genotype is scored for each individual in the pedigree by detecting the (paternally and maternally inherited) allele pair. This allows the inheritance pattern of alleles to be traced through the pedigree structure. Current large scale genotype studies are based on SNP-chip technology, i.e. the identification of bi-allelic Single Nucleotide Polymorphisms, allowing genotypes to be concisely represented by pairing single nucleotide characters (ACGT) or '-' for null sex-linked alleles. Earlier genotype studies used a variety of less automated techniques to assay fewer, often multi-allelic genetic markers (for example, restriction fragment length polymorphisms and microsatellite length polymorphisms). The potential scale of SNP-chip datasets reflects the availability of tens or even hundreds of thousands of SNP markers for study organisms.

Real experimental datasets commonly contain missing genotype data. Whilst this may occur sporadically due to lost samples or failed assays, missing data frequently reflects a systematic decision not to analyse samples for some individuals or generations which may be considered uninformative. The ResSpecies inheritance-checking algorithm infers inherited genotypes for missing data points using the principles of Mendelian inheritance, which may result in either completely resolved or partially resolved (e.g. 'T/?') genotypes. More complex partial inferences are possible for multi-allelic markers (e.g. [T or A]/[T or A or C]) but for simplicity this study only uses bi-allelic markers.

Sex-linked inheritance patterns are observed for markers located on the sex chromosomes, where the heterogametic sex has a single allele for these loci. If sex-linkage is known in advance a null allele may be recorded in a dataset (e.g. 'A/-'), otherwise the genotype would typically be scored erroneously as homozygous (e.g. 'A/A'), with the inheritance pattern of an unrecognized sex-linked marker exhibiting a distinctive error pattern (with many offspring apparently failing to inherit an allele from the heterogametic parent).

### VIPER software

We have previously described *GenotypeChecker*, a simplistic prototype tool for assisting data cleansing [2] that combines the ResSpecies genetic consistency-checking algorithm with a tabular display of genotypes for individuals within a pedigree. The tool highlights inconsistent genotypes and allows the interactive removal of identified erroneous data points. The ability to explore inheritance patterns in a pedigree context is essential for pinpointing the exact data points in error, particularly in the case of incomplete datasets where the inheritance algorithm will infer logically consistent (missing) data from the existing data points and thus 'move' reported errors down to the lowest possible point in the pedigree. Critically, however, the tabular display format does not allow the user to easily explore patterns of errors in the context of the family structures in the pedigree 'tree'. To rectify this situation we have been developing VIPER - Visual Pedigree ExploreR - http://www.viper-project.org.uk/.

The priority of any candidate visualisation to solve our particular problem is to show information such as marker data or error reporting within the context of a pedigree structure. A review of existing techniques for pedigree visualisation showed that current approaches fall short in either one of two categories: scalability and a lack of multivariate data display. Pedigree viewers emanating from a biology background such as PediDraw [3] or Peditree [4] acknowledge and support the integration of biological data on top of a pedigree, but do not scale well, partly because they stick to layout and representation recommendations developed for displaying pedigree data by the biology community [5,6]. Often they are designed not for screen rendering but for outputting hard copy on high definition printers. Unbound by such representation guidelines, approaches to tackle pedigree drawing in the Information Visualisation field have focused on visual scalability through more compact layouts such as PVin and Pedvis [7,8], and through adding interactive features that handle large data sets with operations such as filtering, guided navigating and dynamic zooming like Geneaquilts [9]. However these focus on human genealogies and thus do not accommodate or consider displaying the type of data associated with animal pedigrees such as genotype data. Another specific problem that we recognised was that many pedigree visualisations are set up to show the global structure of pedigrees at the expense of quickly comparing immediate relations e.g.in most of these visualisations to connect between a parent and child involves tracing a link from one part of the screen to another, often through a sea of other links.

**Figure 1:**
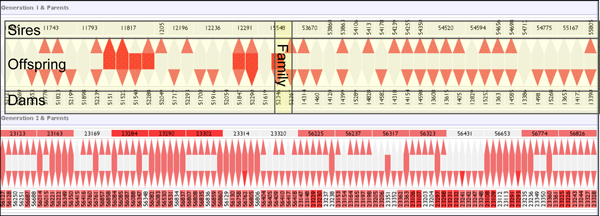
**Screenshot of the sandwich view representation in the VIPER prototype as used in the first evaluation**. Each 'sandwich' shows the relationships between two adjacent generations. The top row shows sires, the bottom row dams, and in between are the offspring. Families are read vertically, cutting across these three rows.

Essentially, we could not find an existing solution that had both the ability to show a pedigree beyond a minimal size, and the ability to show information attached to the individuals within that pedigree. Following a number of design iterations [10] which progressed from initial naive node-link representations, through matrix representations, we finally arrived at the pedigree view seen in Figure 1, which we term the 'sandwich view'. The parent-child relationships between every two consecutive generations is viewed as a sandwich, the male parents (sires) at the top, the female parents (dams) at the bottom, with the children sandwiched in between.

The children are laid out such they lie directly above their female parent and directly below their male parent, making parental relationships much easier to follow on a generation by generation basis. This representation provides the family-centric visualisation necessary to view and assess errors in the context of a pedigree structure.

**Figure 2:**
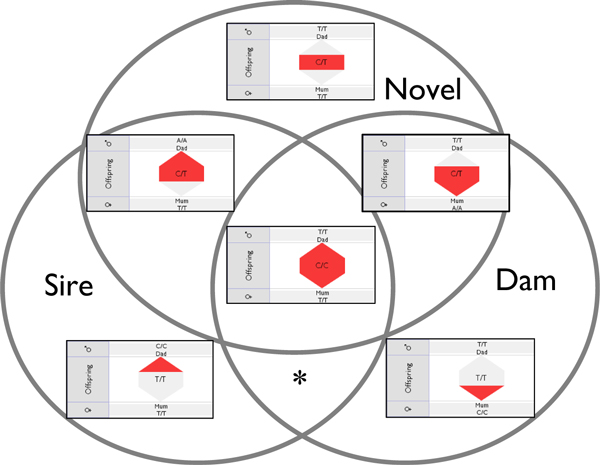
**Six different combinations of possible error for a genotype shown as a Venn diagram**. The only impossible combination of error (marked with an asterisk) is to have errors to sire and dam but to have no novel alleles as well.

Within the VIPER display, a simple discrete four-level colour-coding is used to indicate the proportion of erroneous markers associated with an individual, from white (no errors) through light, mid and heavy colour shading for increasing error rates. Reported inheritance inconsistencies may be categorised into three types: genotypes where no allele is inherited from the sire, where no allele is inherited from the dam, or where a novel, non-parental allele is detected. The three categories of error are represented in the offspring row as sub-parts of a hexagonal glyph, with the tips acting as stylised arrows oriented either up or down. The tips point to the sire and dam rows, colour-coded as described for the number of sire/dam errors for that individual or group across the marker set, and the 'mid-stripe' of the hexagon is similarly coloured for the number of novel allele errors. A single genotype may exhibit any or all of these three error categories, and Figure 2 displays the six different possible combinations of these errors (the seventh combination of errors to sire and dam but no novel allele is impossible). In the sire and dam rows, the combined error count (to sire, dam, and novel allele) for all markers is used to colour the representation of an individual as seen in Figure 1.

**Figure 3:**
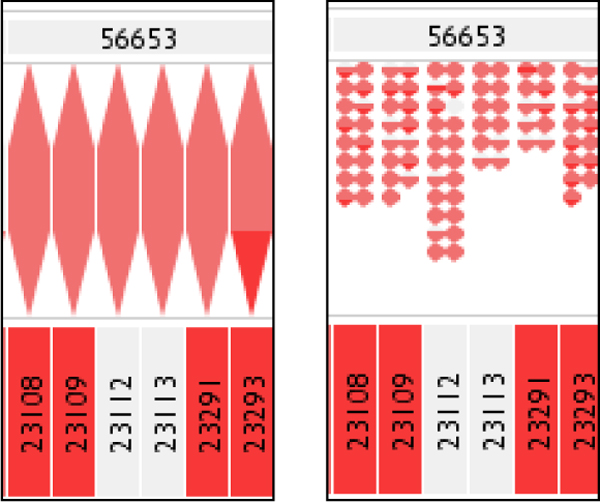
**Aggregated family display (left) compared with individual child display (right)**.

The user can toggle between an aggregated family overview (a single set of statistics per family), or the display of all individuals separately within each family or 'mating pair', as shown in Figure 3. The sandwich views can be ordered from left to right by a selection of metrics, including name, partner count, number of ratio of errors in the offspring groups, and number of errors from offspring to either sire or dam. The offspring sets can be sorted internally by another group of metrics, again including error-related information.

The initial VIPER display looked promising and revealed information about the distribution of errors in the pedigree structure not seen in ResSpecies. Additionally, it also allowed child-parent comparisons that were intractable in other pedigree visualisation tools. However, it still needed much in the way of development in terms of interactivity and specialised biological data display to make it useful for real users with the full range of real data they need to investigate. We therefore embarked on a two stage evaluation of the software, which forms the structure of the rest of this paper. Firstly, an initial evaluation was performed on a small number of test datasets and used to identify and implement any critical features or improvements required for a subsequent functionality evaluation. This second, in-depth evaluation performed by expert biologists tested the effectiveness of the visualisation in helping to identify a variety of representative error states deliberately introduced into simulated pedigree and genotype datasets. We then give an insight into how the size of datasets has scaled since the beginning of the project, along with a description of a complementary node-link visualisation. We then conclude with a discussion of the results and further work.

## Methods

The evaluation was performed in two stages. The first stage validated the ability of VIPER to handle and display the types of datasets to be used in the second stage - a utility evaluation [11] in which we tested the visualisation's ability to faithfully represent pedigree genotype datasets of the necessary scale, and to cope with and indicate errors and omissions within them. This is distinct from usability or efficiency testing, as the primary aim was to ensure the necessary functionality of the system is present. This allowed the identification of critical features for implementation to support data browsing and error localisation, necessary to support the data and events we wished to explore in the subsequent second evaluation.

The second evaluation stage was performed after this extra identified implementation was delivered, and involved testing the visualisation's capability for displaying differing error types commonly found in real-world pedigree genotype datasets. Each separate 'error type' under test was evaluated by creating an appropriate permuted pedigree and genotype data file pair, and then browsing the data visualized in VIPER to verify whether the pattern of inheritance inconsistencies revealed could be used to deduce the underlying, causative error. Simulated datasets were used in order that each error type could be evaluated independently, without the confounding effect of multiple overlapping and interfering errors as found in real datasets.

The two stages of the evaluation were performed by two geneticists from Roslin with extensive experience in analysing and error-cleaning genotyped pedigree datasets. Such an evaluation might not have the numbers of other usability or utility inspection methods, but the expertise of the domain experts in helping assess the visualisation is the overriding factor here [12], any assessment by novices to the domain would be much less informative.

One geneticist created and anonymised the datasets, and monitored the other exploring each dataset in one-to-one sessions lasting an hour. The ease and accuracy with which errors were identified was qualitatively scored, and any issues with the interface or comments about desirable improvements were recorded. These observations formed the basis for deciding which additional information and functionality is essential to present to the user, what modifications might be beneficial but not essential, and any general usability and navigation issues. The approach has a similarity with expert reviews [13] but the experts here are domain rather than visualisation experts, as they are the only ones who can truthfully assess whether the necessary functionality is present and correct.

The majority of the evaluations used a moderately-sized, anonymized chicken pedigree comprising 1792 individuals, the details of which are listed in Table 1.

**Table 1:** Statistics for anonymized chicken pedigree

Generation	Male	Female	Total
F0	28	48	76
F1	16	102	118
F2	0	1598	1598

			**1792**

Alternately pedigrees with controlled numbers of individuals, generations and families per generation were generated de novo using a parameterizable script. Dummy genotype data files for pedigrees were similarly created using a suite of creation and permutation scripts, and desired errors were introduced into either the pedigree or genotype files manually or with further editing scripts.

The simulated genotype datasets reflect the data types found in current large scale studies based on bi-allelic SNPs, including sex-linked markers. A suite of scripts was used to create and then systematically corrupt synthetic genotype data, and to partially erase data from the F1 generation to simulate incomplete data coverage. Initially consistent genotype data was generated using seven different randomly seeded markers. Each marker had bi-allelic SNP alleles C and T, with 5 different C:T heterozygosity ratios (1:1,1:2,1:3,1:4,1:5), 1 mammalian style male sex-linked pair (C, T, Y-null), and 1 female (avian style) sex-linked pair (C, T, W-null). Consistent datasets were generated for 7, 70 and 350 markers by seeding with each marker one, ten or fifty times. The 70 marker genotype dataset proved to be adequate for revealing the expected error pattern for the majority of error types in the data overview.

## Results

### First stage evaluation

We initially validated VIPER's ability to handle and display representative datasets, with regards to four particular aspects of the data in question:

1. The ability to handle pedigrees of a range of sizes.

2. The effect of incomplete data which requires inferring over the missing data.

3. The ability to report systematic errors in sex-linked markers were measured.

4. The ability to restrict the display to the details of a single marker.

This stage of evaluation was spread over three separate hour-long periods, due to the time constraints of the geneticists involved. This did however give an opportunity to correct many minor interface issues they found before they next investigated the prototype, effectively turning the evaluation into a mini RITE-style process [14], of which the who two-stage evaluation process could be considered a larger example. These interface issues included the wish for individual's representations within a generation to be of the same size; previously individuals filled as much space as they could expand into, but the geneticists thought this was giving visual prominence to certain groups, especially families with only a few individuals, leading them to query if they were somehow more important. Also, labelling of sires and dams was switched to display vertically if the space for their display was taller than wide, with a draggable row header allowing the vertical size for such labels to be adjusted (see Figure 3 for an illustration).

### Size and structure of pedigrees

A wide range of animal pedigrees extracted from the ResSpecies data source were tested to confirm the layout and display capabilities of VIPER over a realistic range of experimental pedigrees size and structures. In addition, in order to test the limits of display resolution and usability a number of artificial pedigrees were created as described in the methods section with controlled numbers of total individuals, generations and families per generation, as listed in Table 2.

**Table 2:** Representative results for displaying synthetic large pedigree files.

Individuals	Generations	Families/Generation	Usability Limitations
10 000	30	10	Vertical scrolling accommodates 'any number' of generations.
10 000	3	100	Families display acceptably, but individual offspring render too small for display of genotype labels and clear resolution of error glyphs.
5 000	5	50	Families display acceptably, but individual offspring render too small to display genotype labels.
5 000	3	250	Families at limit of usable resolution for standard monitors, and individual offspring render too small for labeling and error glyph resolution.

Pedigrees were displayed using VIPER on a standard 1280×1024 monitor, and the limits of usable resolution determined, comparing the 'family' with individual offspring views.

In summary, it was demonstrated that the visualisation can cope with any realistic number of generations and over 200 families per generation at the overview level, although the labelling of parent names becomes problematic with over 100 families. However, available space constrains the ability to distinguish the properties of individual offspring where there are a large number of families in a generation (50 to 100) or a large number of offspring in a family. None of the experimental pedigrees available in ResSpecies exceed these thresholds. Display limitations could be ameliorated with higher specification monitors or by expanding across multiple monitors, but in the authors' experience the target user group for VIPER, e.g. animal breeders, often lack high specification desktop hardware and monitors.

It is understood that in future pedigree breeding experiments the number of animals is not expected to get any larger than the figures already seen in ResSpecies or the examples quoted in Table 1. This is because breeding and looking after animals is a resource-thirsty operation requiring real estate and labour to achieve. Even within experiments it is not the case that every animal within a generation goes on to breed, thus avoiding an exponential increase in the number of animals per generation that we would otherwise need to accommodate in the display. The exponential expansion in pedigree genotype data sets will be in the number of markers annotated in the data set as sequencing technology continues to improve. The authors have recently seen data sets containing in the order of 100,000 markers, and this is now not considered large.

### The effect of incomplete data and genotype inference

**Figure 4:**
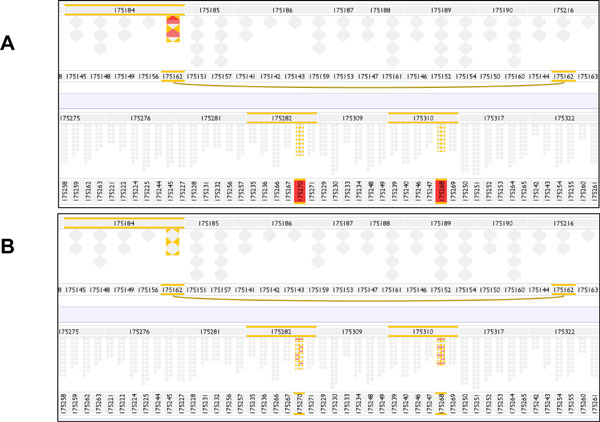
**The effect of incomplete data on error reporting**. In this pedigree two female F1 offspring of female 175162 by male 175216 have been wrongly re-assigned to sire 175184 (in both A and B the two affected sisters and their relations have been highlighted in yellow.) Note that the dam ID 175162 has been duplicated in the dam row of the sandwich because it is now mother to two families, and is connected by an arc underneath the dam row. In (A) the genotype data for the F1 generation is complete, and these two offspring report both multiple failures to inherit from the (incorrect) father and also the occurrence of novel alleles inherited from neither asserted parent. In (B) 50% of F1 genotypes have been erased prior to analysis, and because the algorithm infers incorrect F1 genotypes from the (wrong) parent, errors are revealed between these inferred genotypes and the (correctly identified and genotyped) F2 progeny. Thus the true cause of the errors is obfuscated and the errors are reported in the progeny of the wrongly assigned sisters, rather than in the erroneous sisters themselves.

As described above, in addition to reporting genotypes that are inconsistent with Mendelian transmission, the ResSpecies inheritance algorithm infers missing genotype data by recursively applying allele transmission that must necessarily be true from known data points. As a consequence of the algorithm traversing the pedigree from founders (F0) down through descendants (F1, F2, F3 *etc.)*, errors are reported as low down the pedigree as possible, and particularly in the context of missing data and genotype inference, errors can be reported in individuals (siblings or descendants) removed from the actual source error. The obfuscating effect of this became apparent when synthetic datasets were examined, where a proportion of genotypes were erased from the intermediate generation (F1) individuals, see Figure 4. The 'non-presence' of such missing data would have to be recognised in the visualisation and suitably communicated to users [15].

### Sex-linked markers

**Figure 5:**
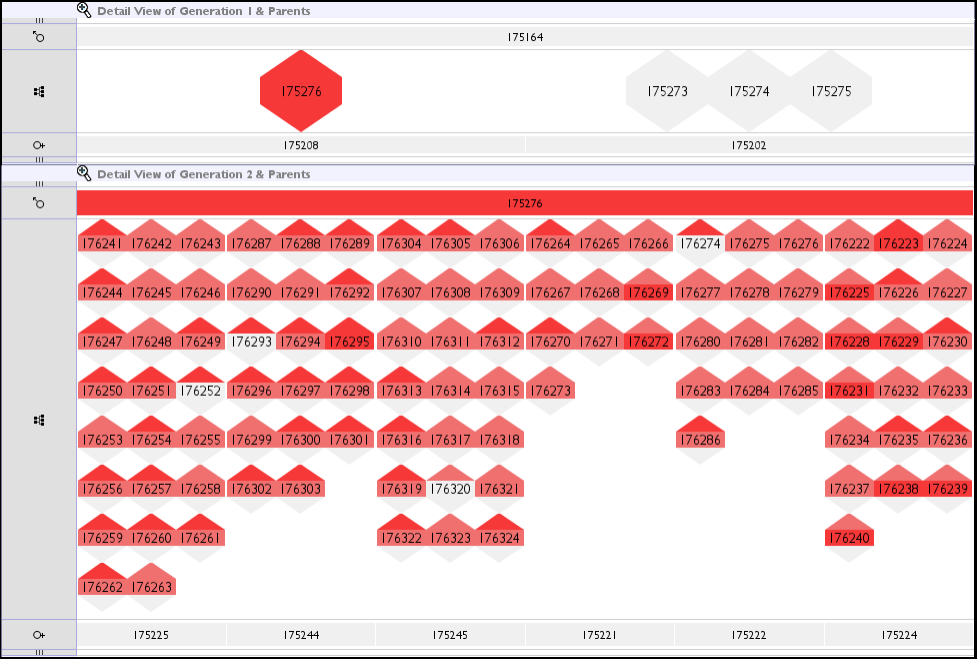
**Sex-linked markers**. Display of a permuted dataset in which multiple sex-linked markers have been altered to be scored homozygous instead of hemizygous. Multiple individuals report 'nil from sire' errors (red upper hexagon). All of the affected individuals are in fact male in this case, and cannot inherit a sex-linked allele from their father. This information is lacking from the interface.

A common systematic error found in real datasets arises when unrecognized sex-linked markers are analysed. Typically this arises in mammals when the genotype assay scores males as homozygous for an allele, whereas in fact they should be heterozygous for the 'Y-null' (absent) allele; the effect is opposite in most birds with heterozygous 'W-null' females unrecognized. As can be seen in Figure 5 this causes a gross systematic error to be reported, immediately apparent as a preponderance of '*nil from sire*' errors (for mammals). However the sex segregation of this effect was not readily apparent because the sex of offspring was not represented in the sandwich view. To readily identify such errors, a method for distinguishing the gender of offspring in the display would have to be developed.

### Single marker view

The geneticists had mentioned the ability to select a single marker and view just its data would be important, and the previous subtleties arising from the sex-linked markers on top of the fact that errors and incomplete data differed from marker to marker reinforced their view. Functionality for displaying the data associated with just a single marker at a time would need to be developed, along with a mechanism for choosing which marker to display. Given that there could be thousands of markers within a pedigree genotype meant this selection would have to be guided by properties of the marker as searching blindly or exhaustively to find markers of interest would be both prohibitive and extremely frustrating.

### Improvements to VIPER

**Figure 6:**
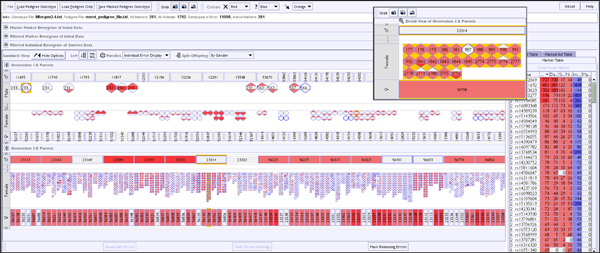
**Improved VIPER interface following the first evaluation step - overview**. Display of the same data analyzed as in Figure 1. Summarised information about markers and individuals is presented in separate tables within a collapsible tabbed pane and can be sorted by error metrics, name, *etc*.; the marker table is used to select a particular marker for display in isolation (see Figure 7). These tables replace the panel in Figure 1 containing visual controls, which are now added to two menu bars. The offspring row can now be separated by gender and the degree of incomplete data per individual is represented with a coloured outline (blue in this case). A floating window holding a detail view of one family is also shown.

**Figure 7:**
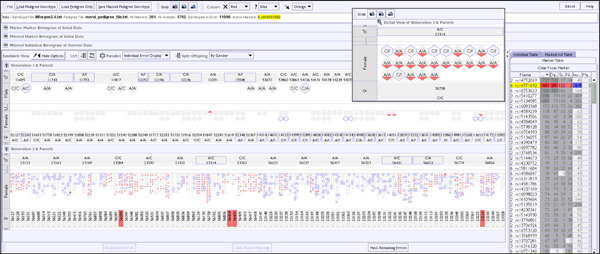
**Improved VIPER interface following the first evaluation step - single marker detail view**. A single marker has been selected for analysis in the dataset shown in overview in Figure 6, and now the actual SNP marker genotype is shown (where space allows). Activation of the 'Detail View' window for a selected family allows inspection of genotypes and errors in full detail. It can also be seen that in the single marker view errors and incomplete data are mutually exclusive, a genotype cannot be both inferred and incorrect.

The initial evaluation of VIPER thus identified several major features which were required prior to performing a full functional evaluation. These improvements are illustrated in the marker summary shown in Figure 6 and in the single marker detail view shown in Figure 7.

In order to support the exploration of individuals in any selected large family a 'Detail View' window was implemented to complement the overview of the entire pedigree 16 (see Figure 7) - one of the standard practices for solving such problems in Information Visualisation [16]. This 'Detail View' can readily accommodate families with hundreds of individuals on a standard monitor, overcoming the resolution constraints identified above.

Secondly, to expose the degree of genetic inference in the data (which occurs because of data incompleteness) a second colour map contrasting with the error colouring was implemented, showing the degree of missing data across the pedigree via the intensity of border colour on an individual or family. As the VIPER display already has space allotted for each individual and family in the pedigree then adding such extra indicators for missing genotype information is not difficult, it would be a different case if there was missing data in the pedigree itself. Clashes between the dual colour highlighting used in the sandwich view to report inference and error rate are limited, because the inheritance algorithm does not infer 'erroneous' genotypes from incomplete data. Control of the colour schemes was provided by adding controls that allow the user to turn off or change the colour used for both the error reporting and the incompleteness maps, and also to choose which contrasting colour is used for user-selection highlighting.

Thirdly, in order to expose the sex of individuals in a family, and hence assist the identification of sex-linked inheritance problems, a further level of colouring was rejected as it would reduce the pre-attentive 'pop-out' [17] that the coloured error display currently enjoyed. Instead the (optional) partitioning of offspring by sex was implemented, spatially separating the male and female offspring into different rows - in effect giving us a 'club sandwich' view. In fact a third, intervening, row was required to accommodate individuals of undetermined sex, as many pedigree datasets include non-breeding individuals that have not been sexed (see Figure 7). In essence, we are using another visual attribute, spatial positioning, to communicate low-count categorical data attributes of the offspring, rather than overload the colour channel. Standard pedigree layouts use shape to represent gender in pedigree diagrams [6] but spatial positioning is a more powerful communicator, especially when we wish to split a set of objects into groups.

In addition to partitioning offspring by gender, the same visualisation mechanism is used to allow segregation of family offspring by other characteristics, such as possession of offspring. This sorting/partitioning mechanism allows the user to explore alternate aspects of inheritance patterns, and will have additional uses once we implement data-cleaning functions that allow modification of various properties of an individual and genotype data.

Finally, the initial VIPER prototype provided only an 'overview' of summary information about inheritance errors averaged across all markers. This summary view adequately exposes many types of systematic errors resulting from wrong pedigree information or sample misidentification, but it does not allow the discrimination of more sporadic errors, nor could the user explore the actual reported genotypes for a given marker. This deficiency was addressed by adding a number of related components to the prototype. Firstly, a sortable 'Marker Table' was added to allow the user to select individual marker genotype data to explore. Markers can be sorted according to their name, counts of reported error types (sire, dam, novel allele or all) and extent of data incompleteness.

A second complementary table for the individuals in the pedigree was also added, with an additional column for gender type. Selection of a named individual highlights it both in the table and the pedigree view, allowing the user to locate individuals of interest. In reciprocation, individuals highlighted in the pedigree view are cross highlighted in the table view. The combination of these two marker and individual information tables not only assists navigation of the data, but provides the detailed error information necessary for deep analysis of inheritance errors. This is augmented by a pop-up tooltip that displays on mouse-over of individuals (or families) within the sandwich view and details the same error information for individuals as reported in the tables. The marker and individual tables use the same colouring schemes for errors and incomplete data as the sandwich view. In this way the sandwich view and tables now form an example of a coordinated multiple view visualisation [18].

Within the marker table, an individual marker could be selected as a 'focal marker', allowing specific genotype information for that marker to be overlaid in the sandwich visualisation - as seen in Figure 7. The single marker pedigree display is essentially identical to the overview, but adds the actual or inferred genotype to the labelling of individuals, allowing the user to analyse in detail the inheritance patterns of alleles in the pedigree.

When data is visualised for a single marker (as in Figure 7), both colouring schemes become binary and mutually exclusive indicators for individual genotypes, as an incomplete data point cannot be marked as an error (the ResSpecies algorithm always assigns a valid value to incomplete data points, even if that assignment is a range of possible values). Thus we are either indicating the presence of an error at that genotype, or that the recorded data was incomplete or absent for an individual genotype, and consequently any genotype shown has been derived by genetic inference, or thirdly no colouring is applied as the genotype is both present and correct. In this mode, the individual table also shows only information for the selected marker, thus indicating for all individuals whether or not each category of possible error occurs.

The addition of these and other controls necessitated the removal of the 'Sort' and 'Display Options' from a dedicated pane area in the first prototype to more traditional menu bar locations. In addition the application now automatically saves a user's layout and colour scheme preferences as default values for the next time the application is run.

With these improvements applied to the VIPER prototype, the second stage of the evaluation proceeded.

### Second stage evaluation

The second stage of the evaluation explored the effect of introducing controlled errors into real and artificial pedigree genotype datasets, and whether they would be represented by the visualisation in a form recognisable to a domain expert.

### Pedigree errors

Real datasets frequently contain errors in the asserted pedigree structure, which might be caused by the misidentification of animals, incorrectly assigned paternity or errors in record keeping. Furthermore sample misidentification or contamination can result in apparent pedigree errors.

In order to evaluate whether the VIPER visualisation adequately exposes the possible kinds of pedigree errors found in real datasets various pedigree disruptions were engineered in the categories given in Table 3. Where appropriate these permutations were performed in separate generations (F0, F1, F2) and upon both same sex and different sex pairs of individuals. Furthermore, to evaluate the potential effects of inference on the observed inheritance patterns, genotype files were derived with 50 or 100% of F1 genotypes erased.

**Table 3:** Categories of pedigree permutations explored in VIPER.

Category	Permutation
1.	Alter father of individual
2.	Alter father of family
3.	Alter father of litter
4.	Alter father of sire sibs
5.	Alter mother of individual
6.	Alter mother of family
7.	Alter mother of litter
8.	Alter parents of individual
9.	Alter parents of family
10.	Alter parents of litter
11.	Alter sex of individuals

All permutations (1-11) were readily identified in the sandwich overview apart from No.11, where inconsistent sex assertions in the pedigree file break the pedigree, causing a fatal error on file loading.

**Figure 8:**
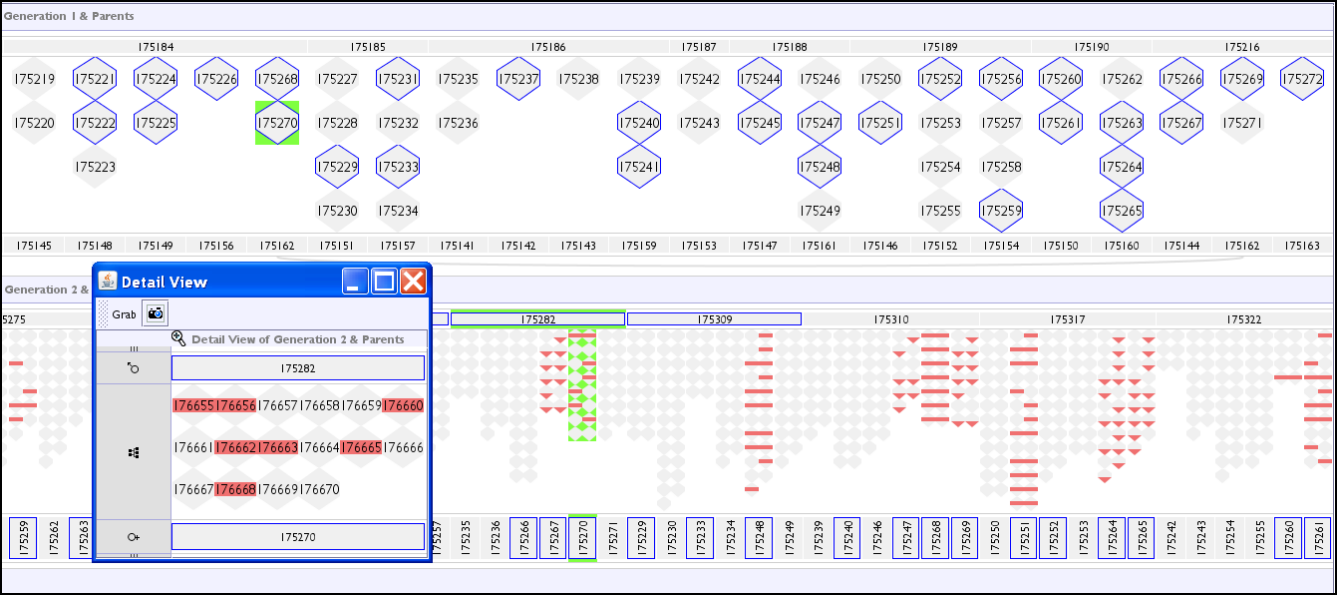
**Highlighting incomplete data**. The same data displayed in the improved VIPER interface as in Figure 4B, in which 50% of F1 are genotypes are deleted. One of the wrongly assigned F1 littermates (175270) is highlighted in green. The addition of blue-border highlighting of incomplete genotype data points throughout the F1 progeny draws the user's attention to the possibility of error propagation by the algorithm. As seen in Figure 4B, the algorithm reports errors in the F2 progeny rather than in the mis-assigned F1 parents. Activating a 'Detail View' for descendants of 175270 shows the reporting of 'novel allele' errors in the F2 offspring.

Pedigree files drawn from the categories in Table 2 were explored in VIPER using the 70 marker test genotype dataset (described above) and with the 100%, then 50% then 0% erased F1 genotypes. The ease with which the error types were located and identified was assessed, and the influence of genotype inference (resulting from missing genotype data) on the inheritance pattern was considered. The categories listed in Table 3 were all successfully explored, with the exception of case 11, which did not pass sanity checking by the ResSpecies algorithm. Permutations with erased genotypes clearly demonstrated the importance highlighting of data incompleteness (with the blue-border), as attention is drawn to the possible obfuscating effect of inference over missing data. As described above (Figure 4) the reported errors are pushed down to F2 when 50 or 100% genotypes are erased, making diagnosis of the root data error more difficult. The improved VIPER prototype draws attention to the lack of genotype data for the wrongly assigned littermates, alerting the user to the possibility that the errors reported in F2 may be propagated from the F1 generation (see Figure 8).

**Figure 9:**
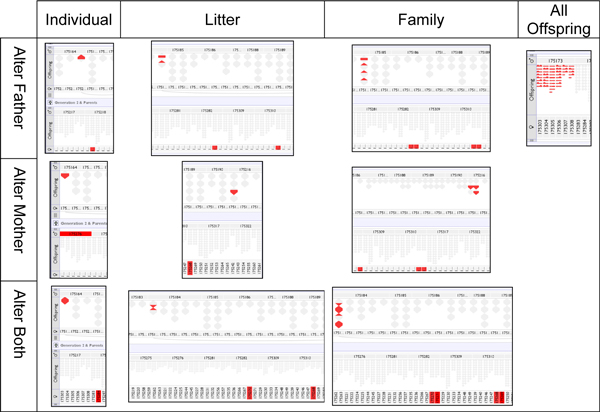
**Grid of all pedigree errors tested with example screenshots**. Sires, dams and then both parents are altered for individuals, litters, families and all offspring (sires only).

Figure 9 shows example screenshots for each of the situations in Table 3 except for "11. Alter sex of individuals", which as reported was caught by the pedigree processing software. Individual changes introduced into the pedigrees manifest themselves as errors in single points in each inter-generation 'sandwich', alterations involving litters show as slightly more colour, and a change to a whole family's parents as slightly more again. Finally, as an example for the sires (as these tend to mate with multiple partners), all of one sire's children have been given the error of belonging to another sire; the resulting glut of errors shows as a cohesive block of colour affecting all the concerned individuals.

### Genotype errors

Genotyping assays can give rise to systematic or sporadic errors. Unreliable assays may give rise to unusable data with very high error frequencies, however a low rate of sporadic 'wrong calls' cannot be discounted for any assay. Errors in sample or data handling may again be systematic or sporadic, and hence might give rise to inconsistency patterns resembling systematic pedigree errors, or to more random, less tractable patterns.

Various types of errors were introduced into hitherto consistent (error-free) genotype files, as categorized in Table 4. Where appropriate, errors were introduced to individuals in different generations (F0, F1, F2), and in order to demonstrate the potential effects of inference on the observed inheritance patterns, alternate data versions created with 50 or 100% of the F1 genotypes erased. The permutations and genotype mixings were also done on both same sex and different sex pairs of individuals.

**Table 4:** Categories of genotype permutations explored in VIPER.

Category	Permutation
1.	Copy (one-way) complete genotypes between individuals
2.	Copy (one-way) some genotypes between individuals
3.	Swap all genotypes from one into a different individual
4.	Swap some genotypes from one into a different individual
5.	Mix some random genotypes into individual
6.	Regenotyped family with novel father
7.	Regenotyped litter with novel father
8.	Regenotyped full sire sib set with novel father
9.	Regenotyped individual with novel father
10.	Score sex linked marker as homozygous
11.	Swap non sibling IDs between generations
12.	Swap non sibling IDs in same generation
13.	Swap sire sibling IDs in same generation
14.	Swap siblings IDs

All permutations (1-14) apart from No.7 were readily identified in the sandwich overview visualisation. Although offspring can be sorted by litter information, there is as yet no suitable visualisation for litter mates; consequently attention is not drawn to errors restricted to a particular litter.

**Figure 10:**
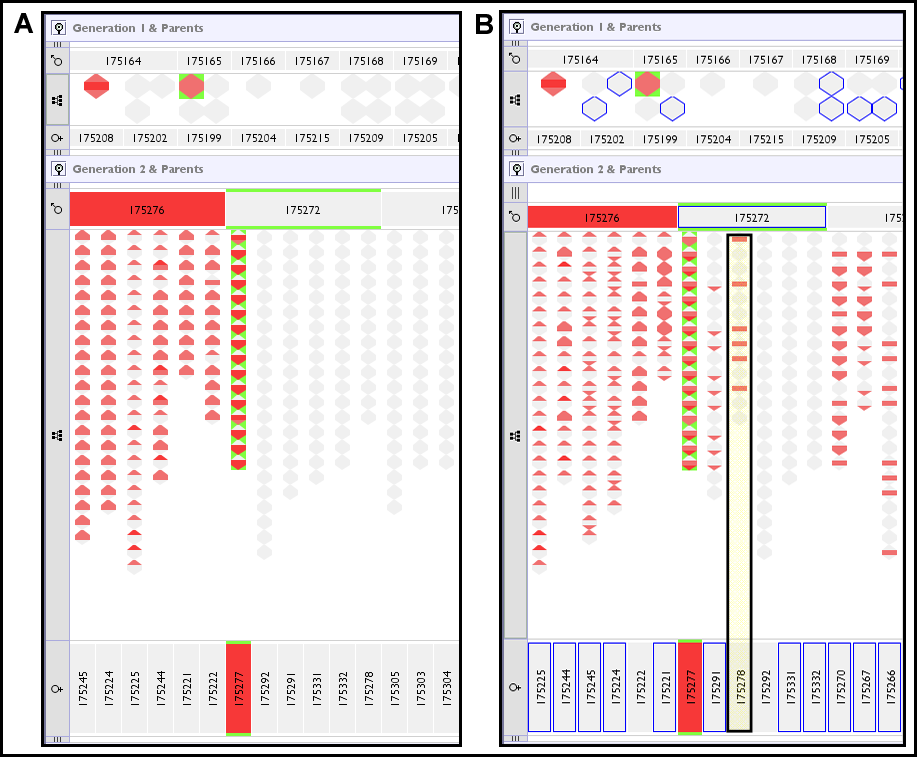
**All genotypes swapped between two unrelated individuals**. (A) Genotype dataset corrupted to swap two samples from generation 1 (175277 female/175276 male). Both samples report multiple inheritance inconsistencies of all three types: nil from sire, nil from dam and novel alleles. Generation 2 offspring from these individuals report novel alleles and failure to inherit from their misidentified parent. In (B) data 50% of the genotype data has been removed from generation 1 individuals (blue borders) causing inference by the genetic algorithm. This has the effect of propagating the reporting of errors through related individuals, for example to offspring of 175278, a sister of the mis-sampled 175277, through inferences on their shared mating partner, the ungenotyped male 175272.

Figure 10 shows analysis of a representative example error of type 1 in Table 4, and models the case where samples (or genotyping results) have been swapped between two unrelated generation 1 individuals, repeated in a genotype with no missing data and with a genotype with 50% of the data removed from F1 individuals. In Figure 10A the inheritance checking algorithm reports multiple apparent inheritance inconsistencies for the misidentified samples, and their offspring. Because the pattern of reported errors is clearly restricted to descendants of the swapped samples, the erroneous data points can be unambiguously resolved. However, when generation 1 genotype data is incomplete (as in Figure 10B), the genetic inference of missing data has the consequence of spreading reported errors through related individuals. In this case errors are propagated through an ungenotyped male partner of one of the 'bad' individuals, to several of his offspring, who are not directly related to the true 'bad' individual. This propagation of errors through incomplete data points obfuscates the identification of the true error source. As real data sets may frequently include incomplete genotype data, often for entire intervening generations, it is important for the user to have their attention drawn to the possibility that the apparent inheritance pattern may be influenced by the data incompleteness.

**Figure 11:**
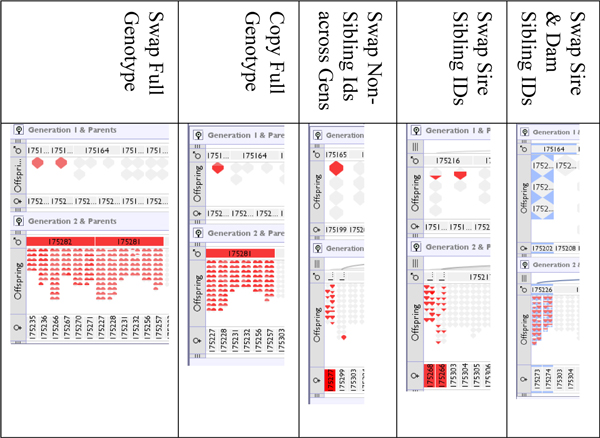
**Screenshots of selected introduced genotype errors for fully complete genotypes**.

It was noted that errors in genotype information tended to result in the display of errors between both the children and parents of the affected individuals. An erroneous genotype in an individual could not be reconciled upwards to one or both of its parents, and in turn that individual's offspring could not reconcile their genotype with the erroneous data in the individual, thus genotype-based errors tended to manifest themselves in two positions. The exception to this was the scenario in which we swapped the IDs of two siblings that shared both parents: in this case both their genotypes could be reconciled upwards as there was no difference in parentage between them, however their offspring reported errors. Example scenarios of introduced genotyping errors can be seen in Figure 11 where the last column shows the case of full siblings being swapped - here we have highlighted the actual swapped IDs of 175273 and 175274 showing that they themselves do not report errors upwards, but their children do.

In the pedigree-based scenarios only the relationship between the affected individual and its incorrectly assigned parents were flagged as error. The individual's offspring were still correctly assigned in the pedigree to the individual. This finding in turn has consequences for the effect of inferencing data and future masking capabilities. Inferencing (i.e. masking) over a wrongly assigned individual in the pedigree will just push the errors down to that individual's children as they cannot reconcile with their grandparents any better than their parent could. However, inferencing over a bad genotype will often remove the error as the individuals in either direction will reconcile once the bad genotype in between is removed.

In summary, identification of these various systematic ID/genotype/parentage swaps proved readily tractable for experienced geneticists using the sandwich pedigree layout. In particular the ability to select and highlight an individual and its ancestors and descendants allows inheritance patterns to be traced.

### Further informal evaluation

The VIPER tool, complete with data masking capabilities, was demonstrated at a meeting of poultry genetic researchers in the UK after the completion of the functional evaluation phase. We found that they appreciated the importance of cleaning the data, and it was revealing that individual's comments focused on the ability to not just mask data but to "correct" data, e.g. to automatically find "correct" parents for misplaced individuals. Similarly, feedback from a sole researcher working at a commercial animal breeder showed that they again quickly zeroed in on wanting to be able to suggest reorganisations of the pedigree and not just masking of erroneous information, however they did view the rest of VIPER's functionality as useful. This is perhaps evidence of a drawback to relying on just the two experts as we did in the evaluation, or simply in a differing definition of what data cleaning entails i.e. making the data good enough to run without error vs. making the data as accurate as possible.

Though VIPER itself is not designed to accommodate any further analyses beyond the cleaning of data i.e. to make it error-free within the constraints of Mendelian inheritance laws, this could be an indication that any next stage in tools for pedigree genotype processing would be for the development of a suite of co-operating tools covering many processes, with data cleaning as just one necessary initial step.

### Large genotype data files

**Figure 12:**
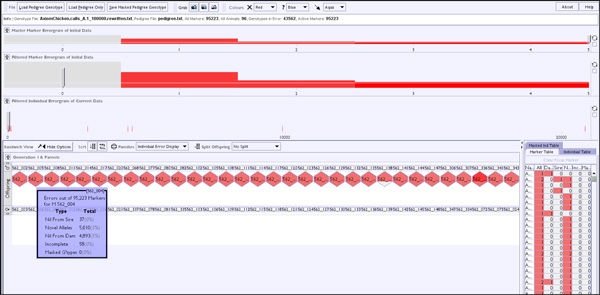
**Example of 100-Kilo marker data set**. For these large marker sets the pedigree is often reduced to a set of 'triples' - unrelated mother-father-child collections.

Extremely large genotype files are now becoming commonplace in bioinformatics; as mentioned earlier in the paper this has meant a step-up from a few thousand markers to data sets with 100,000 markers or more in the pedigree genotype domain. However, as discussed, the VIPER sandwich view and associated histogram views aggregate marker information across the interface and so such an expansion in data is not detrimental to the visualisation, the breaking point is actually when the amount of data loaded overwhelms the available memory. The data set shown in Figure 12 consists of 97 animals over nearly 100,000 markers, making for just less than 10 million data points. Currently this results in the host Java process taking just short of 400 MB of memory.

Individual markers, being separable from other markers, are computed in a time independent of the overall marker set size. When an event affects an individual across all markers then recalculation will take a time proportionate to the marker set size, but for the data set described above this recalculation takes around half a second in total on a 2.83 Ghz PC so is not prohibitive to interaction. VIPER is not concerned with any notion of dividing the marker collection into sets based on segregation patterns or linkage, instead treating each marker as an independent entity operating on top of a shared pedigree. This viewpoint is taken as VIPER is concerned with cleaning the data set which is then passed downstream for further processing. At this point other tools can then perform analyses on top of the data to decide relationships between markers.

Alongside this development some of the 'pedigrees' supplied are now simply collections of triples; sets of father, mother and child unrelated to other triples. This does not result in particularly interesting visualisations but VIPER is still able to display such information.

## Conclusions

The VIPER prototype has been evaluated for the display, exploration and identification of errors in genotyped pedigree datasets, using a range of synthetic datasets which incorporate a wide variety of pedigree and genotype errors, and introduce degrees of data erasure to mimic data incompleteness. The first evaluation exposed a number of critical features that were implemented prior to the full functional evaluation: a 'Detail View' for large families, the colour map of genotype 'incompleteness', the ability to partition siblings by sex and other properties, the marker and individual data tables, and the single marker view functionality. The results of the second functional evaluation confirmed the ability to discriminate the vast majority of single error types in pedigree and genotype datasets.

Findings from the evaluations can be split up into two categories: what we learnt about VIPER in particular and what we discovered about the process of testing an application with domain experts in this manner. For VIPER in particular, the space-efficient layout of the pedigree population in generational layers, organized by mating pairs (families) allows realistically large pedigree datasets to be explored, and the ability to toggle between a summary family view and detailed view of individual offspring provides a workable compromise between a simplified overview and individual detail. The ability to split offspring into categories such as gender also revealed more information than is possible with existing interfaces to this data.

In general, we found that testing the application with datasets of the size and complexity that crop up in the everyday working practices of these domain experts was essential; it validated that the visualisation could cope with data it could expect to encounter in practice. Not having a visualisation that can cope with representative data would negate most, if not all, of the advantage of later bringing in real users to interact with it. This was shown with the larger data sets that are now becoming available, as the layout design of VIPER means most of the effects of an increasingly large marker set do not impact on the visualisation.

Note that we say representative; as well as having real data in the form of ResSpecies pedigrees, we also generated artificial datasets to test the effect of particular combinations of data size and granularity on the visualisation. These artificially generated datasets have the advantage that we know what they should look like in the visualisation if all goes to plan. Such an approach has precedent not just in visualisation but in pedigree analyses, with [19] similarly introducing errors into otherwise correct genotype pedigree datasets to understand their effects. Trying to analyse whether real datasets have rendered properly would depend on a working knowledge of that particular dataset, which approaches a paradoxical situation when considering that gaining such knowledge of that data is why we wish to visualize it in the first place.

This held true into the second evaluation stage where known errors were introduced into real and generated pedigree genotypes. Again, visualising an existing dataset known to have errors would have required deep knowledge of that particular dataset to see if VIPER was communicating those errors properly. By artificially injecting controlled errors, both pedigree and genotype, into clean datasets we can quickly ascertain whether the visualisation is communicating the presence of error and then, according to the domain expert, whether that communication makes sense. There is also the bonus that such datasets could make useful training datasets for new VIPER users in the future. Once the visualisation has been verified as having the functionality necessary to correctly inform an expert user we can then revert to the "real data and real users" mantra.

We also found that the interaction with the geneticists changed from what was expected to be a user-centred process in which they would comment on or test what was implemented to a more participatory approach in which they became much more involved in the design of the prototypes. This is not perhaps entirely surprising given they were the source of the domain knowledge necessary to construct and successfully test the prototypes, and were also likely to be primary users of the finished tool. As such, it was in their interests to shape it to their specific needs. Similarly, it was also a learning process for the visualisation developers as during the course of the project the domain experts made many necessary corrections to the visualisation developer's view of the data and problem. There would not have been anywhere near the same level of mutual feedback if the users had not been domain experts in their field.

The future challenge is to build a data cleaning application that can cope when these errors occur in proliferation and are layered many times on top of each other, rather than the isolated errors we introduced into data sets here. When this is achieved we will have produced a novel tool that not only accurately and compactly visualises error within pedigree genotype data but allows users to clean the errors from such data.

## Competing interests

The authors declare that they have no competing interests.

## Authors' contributions

AL and JK conceived and lead this study. AL is responsible for the ResSpecies data resource and with TP develops the ResSpecies API. TP created the test datasets and performed user testing with AL as 'expert user'. JK and MG designed the VIPER interface and MG implemented the application. TP, MG and JK wrote this manuscript which all authors have reviewed and approved.
